# Efficacy of different nucleoside analog rescue therapies for entecavir-resistant chronic hepatitis B patients

**DOI:** 10.1186/s12879-021-06554-1

**Published:** 2021-09-06

**Authors:** Jin Shang, Juan Zhou, Huan Liu, Rili M. Ise, You Tu, Jinqiu Ran, Lang Bai, Hong Tang

**Affiliations:** 1 Center of Infectious Diseases, West China Hospital, Sichuan University, No. 37 Guoxue Alley, Wuhou District, Chengdu, 610041 Sichuan China; 2 Department of Laboratory Medicine, West China Hospital, Sichuan University, Chengdu, China

**Keywords:** Hepatitis B virus, Chronic hepatitis B, Antiviral treatment, Entecavir, Tenofovir, Adefovir, Drug resistance, Rescue therapy, Virologic response, Renal safety

## Abstract

**Background:**

Entecavir (ETV) is recommended as a first-line anti-HBV treatment. However, many chronic hepatitis B patients initiate anti-HBV treatment such as lamivudine and telbivudine with low genetic barriers in China, which leads to compensatory mutations and increases the rate of ETV resistance. The management of ETV resistance in China is an essential clinical issue.

**Methods:**

Patients from 2011 to 2017 with nucleos(t)ide analog resistance were screened and 72 patients with ETV resistance were included. These patients received different rescue therapies including an ETV and adefovir (ADV) combination therapy group (n = 25), a tenofovir (TDF) monotherapy group (n = 27), and an ETV and TDF combination therapy group (n = 20). Virologic, biochemical, and serologic responses were compared among the three groups.

**Results:**

The rate of ETV resistance among all HBV-resistant variants increased from 6.04% in 2011 to 15.02% in 2017. TDF monotherapy and TDF combination groups showed similar rates of negative HBV DNA at 48 weeks (74.07% vs 70.00%, P > 0.05), while the ETV and ADV group showed the worst virologic response (28.00%). Also, TDF monotherapy and TDF combination therapy showed similar decline of HBV DNA at weeks 12, 24, and 48. There was no significant difference in the rates of HBeAg clearance, ALT normalization, and abnormal renal function among the three groups.

**Conclusions:**

TDF monotherapy showed a comparable virologic response to TDF and ETV combination therapy and a better virologic response than ETV and ADV combination therapy. Thus, TDF monotherapy is the preferred rescue therapy for ETV resistance.

**Supplementary Information:**

The online version contains supplementary material available at 10.1186/s12879-021-06554-1.

## Background

The World Health Organization estimates that 257 million people around the world are currently infected with hepatitis B virus (HBV) and approximately 63 million new cases will occur between 2015 and 2030 [1]. Management of HBV infection remains a global public health challenge. At present, curing HBV is challenging in most patients and they need long-term antiviral treatment. Entecavir (ETV) is recommended as the first-line antiviral treatment in the APASL, AASLD, and EASL guidelines, and the drug resistance of ETV is only 1% over 5 years in treatment-naive patients [2–4].

It is reported that approximately 81% of chronic hepatitis B (CHB) patients receive antiviral drugs with low barrier to resistance such as lamivudine (LMV) and telbivudine (LDT) or adefovir (ADV) before ETV treatment in China [5]. The rate of ETV resistance could increases to 51% in LMV-resistant patients, because if primary LMV resistance mutations occur, compensatory resistance mutations to ETV may arise even if primary LMV treatment is stopped [6]. Drug-resistant patients have higher rates of hepatitis flares and disease progression [7]. Therefore, management of ETV resistance has become an essential clinical issue in China.

Among rescue therapies for patients with ETV resistance, according to the APASL, AASLD, and EASL guidelines [2–4], for patients with only ETV resistance, switching to TDF is recommended, while in patients with multi-drug resistance, TDF or a combination of ETV and TDF are recommended. Because TDF has a high barrier to drug resistance [8], both TDF monotherapy and TDF combined with ETV showed high virologic responses in patients with ETV resistance [9]. Moreover, HBV variants of ADV resistance are also not cross-resistant to ETV, so ETV and ADV combination therapy could be considered in theory, and data in a recent report supported this view [10]. Combining ETV and ADV is also recommended in the APASL guidelines. However, the AASLD and EASL guidelines do not recommended combining ETV and ADV as ETV-resistant rescue therapy. Many patients receive ADV treatment in China because of its relatively low cost. ETV and ADV combination therapy is an alternative rescue therapy for ETV-resistant patients according to the Chinese CHB guidelines by the Chinese Society of Infectious Diseases and Chinese Society of Hepatology. Due to the lack of data on comparative research into ETV–ADV combination therapies to TDF monotherapy or TDF–ETV combination therapy, whether combination ETV and ADV therapy has a comparable efficacy with TDF or TDF plus ETV is worth evaluating. Therefore, this study compared the efficacy of ETV plus ADV combination therapy, TDF monotherapy, and ETV and TDF combination therapy in ETV-resistant patients.

## Methods

The study protocol was approved by the ethics committee of West China Hospital at Sichuan University. Patients were included from the Center of Infectious Diseases, West China Hospital, from 2011 to 2017. Written informed consent was obtained from each patient or his/her legal guardian. Patients with persistent HBV viremia (persistent HBV DNA > 100 IU/ml after 48 weeks of antiviral treatment in plasma) or virologic breakthrough (an increase in HBV DNA levels ≧ 1 log IU/mL in patients who initially responded to antiviral therapy and are compliant with therapy) were eligible for enrollment at screening. Resistance mutations were determined by direct sequencing of the reverse transcriptase region of the HBV polymerase gene (pol/RT). Patients with confirmed ETV genotypic resistance mutations (the presence of rtT184A/C/F/G/I/L/S, rtS202G, or rtM250L/V, in addition to L180M + M204V/I mutation) were included.

Patients with underlying liver diseases such as non-HBV viral hepatitis, nonalcoholic fatty liver diseases, and autoimmune hepatitis were excluded. Patients with underlying severe chronic respiratory diseases, cardiovascular disease, and chronic kidney injury were not enrolled. Patients lost to complete follow-up were also excluded. Ultimately, 72 patients with ETV resistance (rtL180M, rtT184A/C/F/G/I/L/S, rtS202G, rtM250L/V, and rtM204V/I) combined with ADV resistance (rtA181V/T and/or rtN236T) were included. The patients switched to three rescue therapies, including ETV/ADV combination therapy, TDF monotherapy, and ETV/TDF combination therapy. After 48 weeks of rescue treatment in patients with ETV resistance, parameters including HBV DNA levels (ranging from 100 IU/mL to 5 * 107 IU/mL), HBV serological markers (HBsAg, antibody to HBsAg, HBeAg, antibody to HBeAg, antibody to HBcAg), liver function (TB: total bilirubin, DB: direct bilirubin, IB: indirect bilirubin, ALT: alanine aminotransferase, AST: aspartate aminotransferase, TP: total protein, ALB: albumin, GLB: globin, GGT: gamma-glutamyl transpeptidase, TBA: total bile acid), renal function (urea, creatine, uric acid, Cys-C, eGFR) were analyzed. In addition, the virologic response, rate of normal alanine aminotransferase (ALT), and incidence of HBeAg loss/seroconversion were compared in the three groups. Adverse events were also assessed throughout 48 weeks. The glomerular filtration rate was estimated using the modification of diet in renal disease equation as follows: estimated glomerular filtration rate (eGFR; milliliters per minute per 1.73 m^2^) = 186 * serum creatinine^−1.154^ * age^−0.203^ * (0.742 if female) * 1.233 (Chinese) (Additional file 2: Figure S1).

### Statistical analysis

Group–group comparisons of continuous variables were conducted using the analysis of variance, t test, Chi-squared test, or Fisher’s exact test. All of the statistical analyses were conducted using SPSS version 22.0. P < 0.05 was considered statistically significant.

## Results

### Trends in ETV resistance mutations

**Fig. 1:**
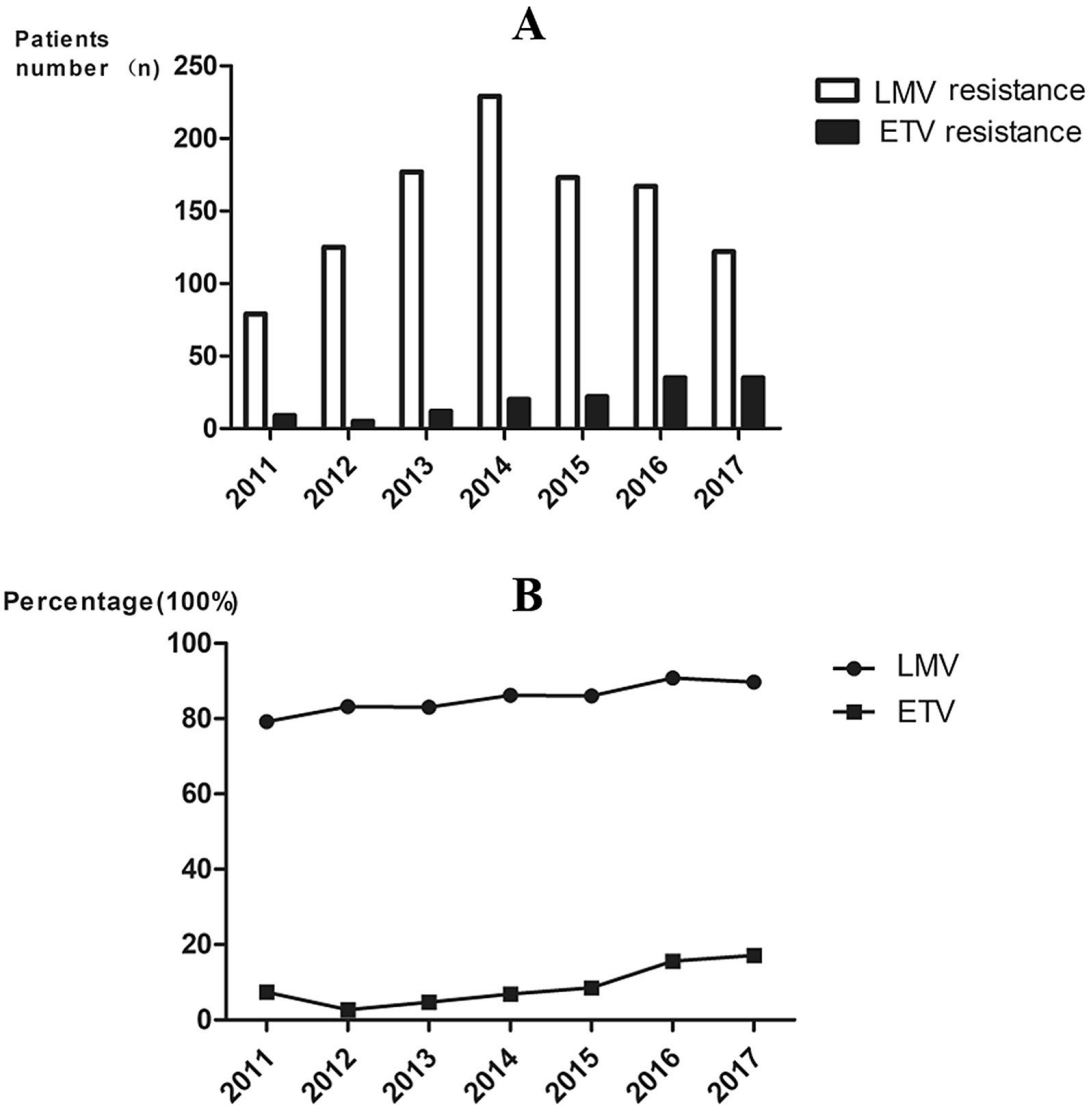
Trends of nucleos(t)ide analogue resistance mutations. Occurrence rate of ETV resistance varies from 2011–2017, **A** Number of patients with LMV resistance and ETV resistance; **B** Proportion of LMV resistance and ETV resistance among all patients with nucleos(t)ide analogue resistance

The rates of ETV resistance gradually increased from 6.04% in 2011 to 15.02% in 2017, and the proportion of LAM/LdT resistance was high from 2011 to 2017. Overall, 166 patients with ETV resistance were screened and 72 were included (Fig. 1 and Additional file 2: Figure S1).

### Baseline characteristics of patients with ETV resistance

The 72 patients in the ETV/ADV (n = 25), TDF (n = 27), and ETV/TDF groups (n = 20) completed 48 weeks of treatment. The median age was 43, 44, and 46 years in the ETV/ADV, TDF, and ETV/TDF groups, respectively. The proportion of males was 80.00%, 85.19%, and 65.00% in the ETV/ADV, TDF, and ETV/TDF groups, respectively. The median HBV DNA level was 6.31, 5.40, and 6.23 log10 IU/mL, respectively. Median duration of previous LMV treatment before ETV treatment was 28 (6–110) months in ETV + ADV group, 26 (6–64) months in TDF group, 26 (6–72) months in TDF + ETV group. No significant differences occurred in the age distribution, male proportion, baseline HBV DNA level, duration of previous LMV treatment or other liver function, and renal function tests (Table 1).

**Table 1:** Baseline characteristics of patients with ETV-resistance in different rescue therapy groups

	ETV + ADV	TDF	TDF + ETV
N	25	27	20
Age, years	43 (21–64)	44 (20–64)	46 (30–68)
Male, n (%)	20, 80.00%	23, 85.19%	13, 65%
ALT (IU/L)	120.46	61.12	71.00
Normal ALT, n (%)	13, 52.00%	17, 62.96%	10, 50.00%
Bilirubin, mg/dL	18.93	20.67	33.86
Creatinine, mg/dL	78.97	90.62	75.43
HBeAg-positivity, n (%)	17, 67.00%	23, 85.19%	15, 75.00%
HBV DNA (log10 IU/mL)	6.31	5.40	6.23
Previously exposed NUC, n			
LMV	23	14	14
ADV	0	6	3
LDT	1	2	1
LMV + ADV	1	5	2
Duration of NUC treatments (months)	28 (6–110)	26 (6–64)	26 (6–72)
Resistance mutations			
ETV	18	22	17
Multidrug resistance	7	5	3

### Virologic, biochemical, and serologic responses in the subgroups

**Fig. 2:**
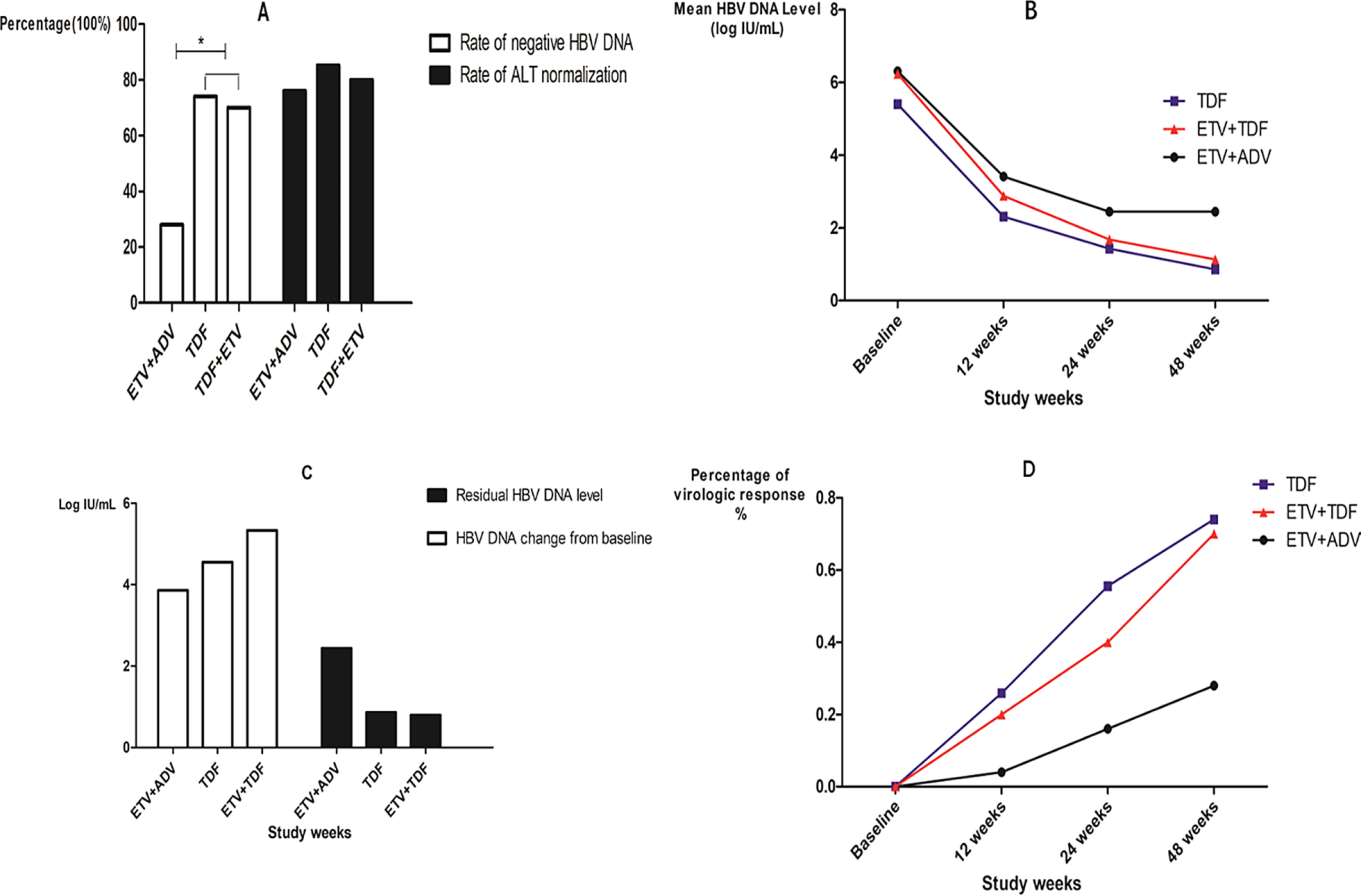
Virologic, biochemical, and serologic responses of different rescue therapy. Virologic response, biochemical response and serologic response in three groups were analyzed, **A** Rate of negative HBV DNA and rate of ALT normalization in different rescue therapy groups. **B** Mean HBV DNA level decline at 12, 24, 48 weeks in different rescue therapy groups. **C** Residual HBV DNA and HBV DNA change from baseline level in different rescue therapy groups. **D** Percentage of virologic response in different rescue therapy groups

Three patients in the ETV/ADV group discontinued because of poor virologic response and switched to ETV/TDF combination therapy. One patient in the TDF group discontinued because of risk of renal injury. Among the patients in the TDF and ETV/TDF groups, no significant difference was found in the rate of achieving virologic response at 48 weeks (74.07% vs 70.00%). The mean level of HBV DNA significantly decreased at 12 weeks, 24 weeks, and 48 weeks, and no virologic breakthrough occurred in these two groups. However, the ETV/ADV group showed a significantly low rate of achieving virologic response at 48 weeks (28%). The residual HBV DNA level was also significantly higher than in the TDF or ETV/TDF groups. Virologic breakthrough occurred in one patient after 40 weeks of ETV/ADV treatment. The patient switched to ETV/TDF, and their HBV DNA declined to negative after 12 weeks of treatment. As for serologic responses, HBeAg seroclearance occurred in 3 patients in the TDF group, 1 patient in the ETV/TDF group, and 0 patients in the ETV/ADV group, with no significant difference (Table 2 and Fig. 2). To evaluate whether poor virologic response in the ETV/ADV group was due to the relatively higher rate of multi-drug resistance, we conducted a subgroup analysis of ETV resistance without ADV resistance. We found that in the patients with only ETV resistance, the ETV/ADV group still demonstrated a significantly lower rate of virologic response at 48 weeks, and the residual HBV DNA level was higher than in the other two groups (Table 3).

**Table. 2:** Virologic, biochemical, and serologic responses of ETV-resistance patients with/or without ADV resistance in different rescue therapy at Week 48

	ETV + ADV	TDF	TDF + ETV
N	25	27	20
HBV DNA < 100 IU/mL, n (%)	7, 28.00%	20, 74.07%	14, 70%
HBV DNA change from baseline (log10 IU/mL)	3.86 ± 1.62	4.55 ± 1.81	5.33 ± 1.88
Residual HBV DNA level (log10 IU/mL)	2.44	0.86	0.8
Virologic breakthrough	1	0	0
ALT (IU/L)	40.56	45.33	34.82
ALT normal, n (%)	19, 76.00%	23, 85.19%	16, 80.00%
Abnormal renal function	0	1	1
HBeAg seroclearance, n (%)	0	3	1
HBeAg seroconversion, n (%)	0	1	1
HBsAg seroclearance, n (%)	0	0	0
Discontinued	3	1	0

**Table. 3:** Subgroup analysis of virologic, biochemical, and serologic responses of patients with only ETV resistance different rescue therapy at Week 48

	ETV + ADV	TDF	TDF + ETV
N	18	22	17
HBV DNA < 100 IU/mL, n (%)	5, 27.28%	15, 68.18%	11, 64.71%
HBV DNA change from baseline (log10 IU/mL)	4.09	4.07	5.03
Residual HBV DNA level (log10 IU/mL)	2.45	1.19	1.03
Virologic breakthrough	0	0	0
ALT (IU/L)	41.44	44.1	35.13
ALT normal, n (%)	13, 72.22%	19, 86.36%	14, 82.35%
Abnormal renal function	0	0	1
HBeAg seroclearance, n (%)	0	3, 13.64%	1, 5.88%
HBeAg seroconversion, n (%)	0	1, 4.55%	1, 5.88%
HBsAg seroclearance, n (%)	0	0	0
Discontinued	3	1	0
12 weeks HBV DNA level (log10 IU/mL)	3.75	2.47	2.83
24 weeks HBV DNA level (log10 IU/mL)	2.44	1.36	1.84
48 weeks HBV DNA level (log10 IU/mL)	2.45	1.19	1.03
12 weeks HBV DNA < 100 IU/mL, n (%)	0.00%	27.27%	17.65%
24 weeks HBV DNA < 100 IU/mL, n (%)	16.67%	54.55%	41.18%
48 weeks HBV DNA < 100 IU/mL, n (%)	27.78%	68.18%	64.71%

### Safety profiles

Regarding the safety of the different treatments, only 1 hepatocellular carcinoma occurred in the ETV/ADV combination group, and no ALT flares occurred in either group. No significant difference occurred in the discontinuation rate due to adverse events in the three groups. As for eGFR measurement, differences in the baseline eGFR level, eGFR at 48 weeks, and eGFR decline from baseline or the rate of eGFR < 90 mL/min/1.73 m^2^ at 48 weeks demonstrated no significant differences (Table 4 and Additional file 3: Figure S2). Other detailed parameters were all showed in the raw data (Additional file 1).

**Table. 4:** Safety Profiles of patients in different rescue therapy group at 48 weeks

	ETV + ADV	TDF	TDF + ETV
Patient number	25	27	20
HCC	1	0	0
Cirrhosis	0	0	0
Deaths	0	0	0
ALT flare	0	0	0
Discontinuation due to adverse event	2	1	1
baseline eGFR	125.82	122.75	127.71
eGFR at 48 weeks	103.76	112.32	110.26
eGFR decline from baseline	22.06	10.43	17.45
eGFR < 90 mL/min/1.73 m^2^ at 48 weeks, n(%)	3, 12.00%	1, 3.70%	3, 15.00%

## Discussion

From 2011 to 2017, the rate of ETV resistance among all HBV-resistant variants increased from 6.04 to 15.02%. ETV resistance is becoming severe in China since antiviral drugs such as LMV, LDT, and ADV with low barriers to resistance are commonly used, and the number of CHB patients in China constitutes approximately one-half of the global CHB population [11]. As for the mechanism of ETV resistance, ETV resistance barrier is lower by initial selection of LMV-resistant HBV mutation. The primary mutations of LMV resistance are the methionine residues at amino acid 204 conferred to isoleucine or valine M204 I/V, while methionine or serine changes at 180 (rtL180M/S) usually accompany this [12]. Once such primary resistance mutation occurred, the ETV resistance, which needs a mutation at B domain (rtI169T or rtS184G), C domain (rtS202G/I), or E domain (rtM250V) on basis of rtM204V/I ± rtL180M/S mutation, is much more likely to achieve [13]. Therefore, because of a large accumulation number of LMV resistance, the rate of ETV resistance increased during the long-term course in NA-experienced patients, which indicated that monitoring the resistance of ETV requires more attention.

Regarding the efficacy of different rescue therapies for patients with ETV resistance, the combination of ETV and TDF has potential benefit on minimizing the risk of potential mutations and improving the antiviral efficacy during the TDF rescue therapy. In our study, TDF and TDF plus ETV combination therapy showed comparable virologic response at 24 or 48 weeks, which was similar to previous studies on patients with partial virologic response to ETV [14, 15]. In multi-drug-resistant CHB patients, TDF also demonstrated comparable efficacy and safety to TDF plus ETV combination therapy [16]. Theoretically, mutations such as N236T and A194T are potential variants with resistance to TDF, which has no cross-resistance to ETV. TDF monotherapy is likely to have comparable antiviral effects compared with TDF plus ETV combination therapy. However, one major concern is that whether TDF genetic resistance occurred in LMV-experienced patients [17]. It is reported that rtL180M/T184L/A200V/M204V mutation with resistance to TDF was found in ETV-resistant patients receiving TDF monotherapy [18]. Whether primary resistant mutations to ETV resistance could increase the rate of mutations resistant to TDF in long-term TDF monotherapy is unknown. Long-term clinical trials on TDF and TDF plus ETV combination therapy demonstrated that no persistent HBV viremia or virologic breakthroughs occurred in TDF monotherapy at 144 weeks or 240 weeks [16, 19]. In an in vitro study, TDF was also susceptible in both LMV-resistant clones and ETV-resistant clones [20]. Therefore, we hypothesis the combination of ETV and TDF didn’t have better antiviral efficacy and lower risk of potential mutations resistance than TDF monotherapy.

Regarding the safety profile of the TDF monotherapy group and TDF plus ETV groups, the baseline eGFR and eGFR at 48 weeks were comparable, no significance exists in renal safety between these two groups. As for the potential mechanism of renal toxicity of TDF, TDF is excreted via glomerular filtration, and active tubular transport and may cause proximal tubular dysfunction [21]. Which was not found in ETV treatment. However, there was no evidence that ETV has renal protective effect, also a combination of ETV and TDF didn’t show better renal safety than TDF in previous researches [22, 23]. Therefore, we posit that no difference in renal safety exists between TDF monotherapy and TDF plus ETV combination therapy. Consider that long-term adherence and the cost-effectiveness of monotherapy is better than combination therapy, TDF monotherapy could be optimal treatment strategy for patients with underlying renal or bone metabolism diseases.

The virologic response of ETV plus ADV combination therapy is the worst among the three therapies regardless of multi-drug resistance. Only 28% of patients achieved virologic response, and 72% of patients had persistent HBV viremia at 48 weeks. Totally, three patients in ETV + ADV group discontinued the rescue therapy due to poor antiviral efficacy. They all switched to TDF monotherapy. One patient achieved virologic response 12 weeks after switching to TDF and another patient achieved virologic response 24 weeks after switching to TDF. The 3rd patient’s HBV DNA load reduced to 6.76E + 03 IU/mL 48 weeks after switching to TDF, who suffered decompensation of liver cirrhosis for three times during this period. Therefore, more effective rescue therapy should be selected to avoid persistent viremia, which is associated with a disease progression in long-term treatment. Although major ADV-resistant mutations were rtA181V/T mutations and rtN235T without cross-resistance to ETV, the relatively weak antiviral efficacy of ADV limits its use in ETV-resistant patients. In previous research, ADV had an estimated 30% resistance rate after 5 years of treatment in LMV-resistant patients [24]. Moreover, ADV and LMV dual resistant mutations may occur in LMV-resistant patients receiving ADV rescue therapy [25]. Additionally, TDF is easy to access and extremely inexpensive because of the new government procurement policy in China. ETV and ADV combination therapy has lower antiviral efficacy without the advantage of safety or cost-effectiveness. Therefore, TDF monotherapy may be the preferred rescue therapy rather than ETV and ADV combination therapy.

## Limitation

Since the rate of ETV resistance is relatively low, although we have screened a large cohort including 1837 HBV patients with genomic resistance to antiviral treatment, the number of patients with ETV resistance were limiting.

## Conclusion

ETV resistance has gradually become a severe clinical problem in China because of the large number of antiviral treatment-experienced patients. TDF showed comparable virologic response and tolerance to TDF plus ETV combination therapy and better virologic response than ETV plus ADV combination therapy. TDF monotherapy may be the optimal strategy for CHB patients with ETV resistance.

## Supplementary Information

**Additional file 1.** Some raw data generated or analyzed during this study were showed. Sheet 1 included data in screening process and Sheet 2 included data in dividing group and analyzing process.

**Additional file 2: Figure S1.** Flow diagram of included patients. Flow diagram of including process were showed including 4 steps including screening, excluding, including and dividing to groups.

**Additional file 3: Figure S2.** Renal safety of different rescue therapy groups. eGFR, which reflect the renal function, was analyzed in different rescue therapy groups, (A) Mean eGFR level at baseline and 48 weeks after rescue therapy; (B) Change of eGFR level in different rescue therapy groups.

## Data Availability

The datasets used and analysed during the current study are available from the corresponding author on reasonable request.

## Abbreviations

HBV: Hepatitis B virus
ETV: Entecavir
TDF: Tenofovir disoproxil fumarate
ADV: Adefovir dipivoxil
CHB: Chronic hepatitis B
LMV: Lamivudine
LDT: Telbivudine
