# Supplementary material for: Efficacy of different nucleoside analog rescue therapies for entecavir-resistant chronic hepatitis B patients
Source: BMC Infect Dis. 2021 Sep 6;21:912. doi: 10.1186/s12879-021-06554-1 (PMC8420064; doi:10.1186/s12879-021-06554-1)
Supplement: Supplementary file 1 — Additional file 1. Some raw data generated or analyzed during this study were showed. Sheet 1 included data in screening process and Sheet 2 included data in dividing group and analyzing process. [file 12879_2021_6554_MOESM1_ESM.docx]

Table S1 Trends of hepatitis B virus resistance to nucleoside/nucleotide analogues from 2011 to 2017

|  | 2011 | 2012 | 2013 | 2014 | 2015 | 2016 | 2017 |
| --- | --- | --- | --- | --- | --- | --- | --- |
| Patients(n) | 149 | 220 | 277 | 362 | 315 | 281 | 233 |
| LAM/LdT | 79 | 125 | 177 | 229 | 173 | 167 | 122 |
| ADV | 21 | 29 | 33 | 30 | 34 | 18 | 16 |
| LMV+ADV | 28 | 51 | 40 | 58 | 72 | 45 | 49 |
| ETV | 9 | 5 | 12 | 20 | 22 | 35 | 35 |
| Multidrug resistance | 2 | 1 | 1 | 5 | 5 | 9 | 5 |
