# Supplementary figures and images for: Efficacy of different nucleoside analog rescue therapies for entecavir-resistant chronic hepatitis B patients
Source: BMC Infect Dis. 2021 Sep 6;21:912. doi: 10.1186/s12879-021-06554-1 (PMC8420064; doi:10.1186/s12879-021-06554-1)

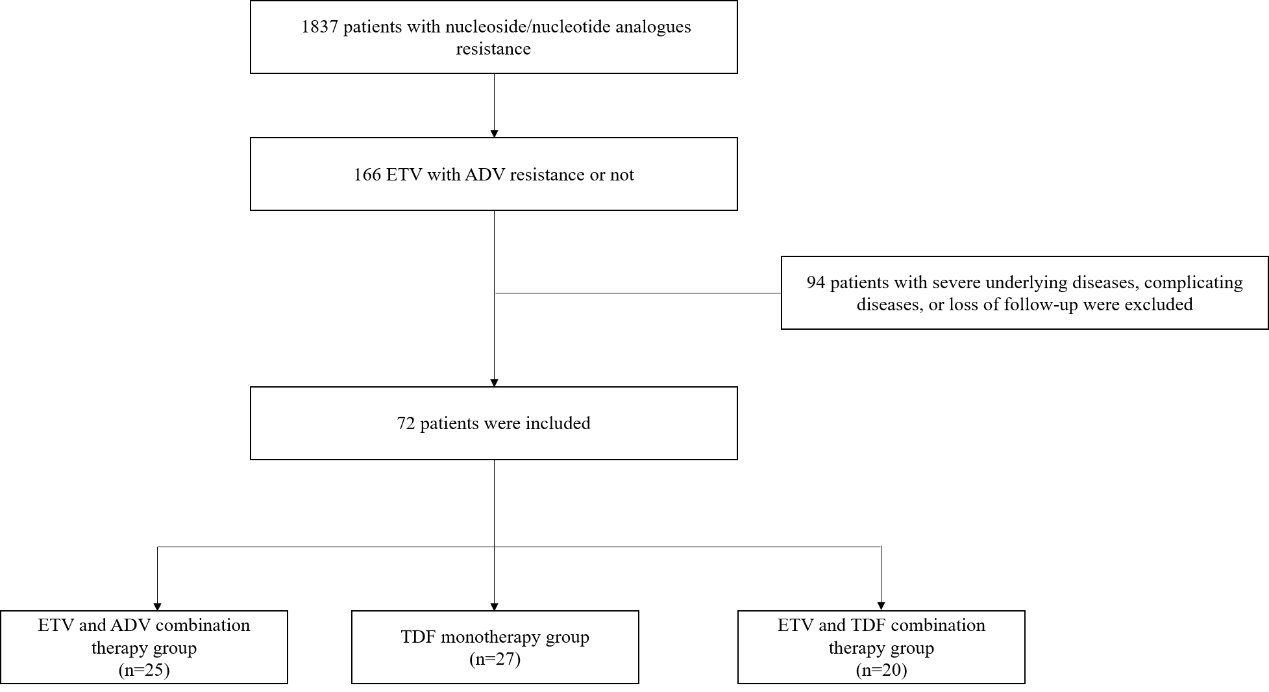

Supplement: Supplementary file 2 — Additional file 2: Figure S1. Flow diagram of included patients. Flow diagram of including process were showed including 4 steps including screening, excluding, including and dividing to groups. [file 12879_2021_6554_MOESM2_ESM.tif]

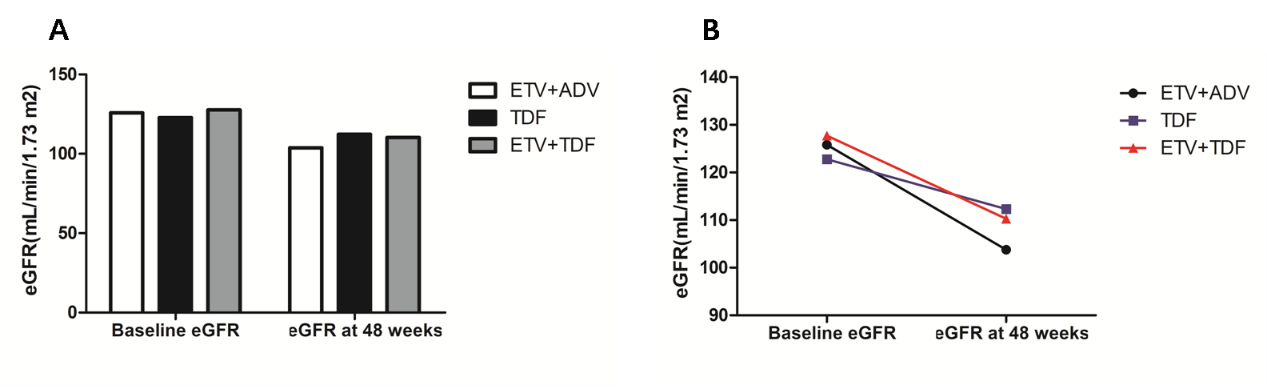

Supplement: Supplementary file 3 — Additional file 3: Figure S2. Renal safety of different rescue therapy groups. eGFR, which reflect the renal function, was analyzed in different rescue therapy groups, (A) Mean eGFR level at baseline and 48 weeks after rescue therapy; (B) Change of eGFR level in different rescue therapy groups. [file 12879_2021_6554_MOESM3_ESM.tif]
